# Evaluating the Effect of Zingiber *Officinalis* on Nausea and Vomiting in Patients Receiving Cisplatin Based Regimens

**Published:** 2011

**Authors:** Fanak Fahimi, Kian Khodadad, Somayeh Amini, Farzaneh Naghibi, Jamshid Salamzadeh, Shadi Baniasadi

**Affiliations:** a Pharmaceutical Care Department, Chronic Respiratory Disease Research Center, National Research Institute of Tuberculosis and Lung Disease (NRITLD), Masih Daneshvari Hospital, Shahid Beheshti University of Medical Sciences, Tehran, Iran.; b Clinical Pharmacy Department, School of Pharmacy, Shahid Beheshti University of Medical Sciences, Tehran, Iran.; c Chronic Respiratory Disease Research Center, National Research Institute of Tuberculosis and Lung Disease (NRITLD), Masih Daneshvari Hospital, Shahid Beheshti University of Medical Sciences., Tehran, Iran.; d Traditional Medicine and Materia Medica Research Center, Shahid Beheshti University of Medical Sciences, Tehran, Iran.; e Pharmaceutical Care Department, Virology Research Center, National Research Institute of Tuberculosis and Lung Disease (NRITLD), Masih Daneshvari Hospital, Shahid Beheshti University of Medical Sciences, Tehran, Iran.

**Keywords:** Chemotherapy, Cisplatin, Ginger, Nausea, Vomiting

## Abstract

Ginger, the rhizome of *Zingiber officinalis*, has long been used as herbal medicine for its antiemetic effect. For evaluating the effect of *zingiber officinalis* on nausea and vomiting (N and V) in patients receiving cisplatin based regimens, a randomized double-blind placebo-controlled cross-over clinical trial was carried out in patients receiving cisplatin in combination with other chemotherapeutic agents. The patients were randomly assigned to receive ginger capsules (rhizome of *zingiber officinalis*) or placebo in their first cycle of the study. All patients received standard antiemetics for chemotherapy induced nausea and vomiting (CINV). The patients were crossed-over to receive ginger or placebo in their next cycle of chemotherapy. Among 36 eligible patients who received both cycles of treatment, there were no difference in prevalence, severity, and duration of both acute and delayed N and V. Addition of ginger to the standard antiemetic regimen has shown no advantage in reducing acute and delayed N and V in patients with cisplatin-based regimen in this study.

## Introduction

N and V are the common complications of chemotherapy in cancer patients (1). Cisplatin, one of the cornerstones in several chemotherapy regimens, is a highly emetogenic chemotherapeutic agent (2). The effect of antiemetic treatments of 5-HT_3_ receptor antagonists, corticosteroids, and metoclopramide both alone and in combination, have been evaluated on delayed N and V induced by cisplatin. Depending on the dose and administration of cisplatin and the combination of antiemetic agents, 21% to 65% of patients may experience delayed nausea and vomiting (3). Ginger, the rhizome of *Zingiber officinalis*, has long been used as herbal medicine for its antiemetic effect. It is still a matter of debate whether the herb is efficacious for chemotherapy induced nausea and vomiting (CINV). Animal studies have shown that ginger is effective for CINV (4, 5, 6) but human studies have revealed diverse results (7, 8, 9, 10). Cisplatin induces N and V by decreasing the rate of gastric emptying and accumulation of food in the stomach, which can cause sensations of bloating, nausea and vomiting. Ginger increases gastric emptying rate through scavenging of free radicals, blocking the 5-HT3 receptors, and decreasing the amount of serotonin in the intestines (5). The current study was designed to evaluate the effectiveness of ginger, when given together with the standard antiemetic regimen for the management of acute and delayed N and V, in patients with cisplatin based chemotherapy regimens.

## Experimental

*Study design, setting and subjects*

The investigation was designed as a randomized, cross-over, double-blinded, and placebo-controlled clinical trial comparing the effect of oral ginger 1 g/day versus placebo on CINV in patients receiving cisplatin-based anticancer regimens. The study was conducted in the oncology ward of the National Research Institute of Tuberculosis and Lung Disease (NRITLD), affiliated to Shahid Beheshti University of Medical Sciences (Tehran, Iran). Ethical permission for the study was obtained from the ethical review board of Shahid Beheshti University. All eligible patients signed a written informed consent prior to their participation in the study.

Adult subjects (age > 18 years) with the following inclusion criteria were entered into the study:

Diagnosis of cancer and receiving cisplatin-based regimens at least for two cycles of the chemotherapy.

Receiveing one of the standard antiemetic regimens [5-HT3 antagonist (granisetrone) and corticostreoid (hydrocortisone)].

Being able to swallow capsules. The following exclusion criteria were applied: Clinical evidence of current or impending bowel obstruction. Current radiotherapy, that is classified as high or intermediate risk of causing nausea and vomiting. Pregnancy or lactation. Concurrent use of ginger products (powder, tea, ...).

Concurrent therapeutic doses of warfarin, aspirin or heparin. Thrombocytopenia or history of abnormal bleeding in the past 6 months. Symptomatic brain metastasis.

Thirty-six participants were randomized to receive 4 capsules of powdered ginger (Zintoma®, Gol Daru) daily (each capsule contained 250 mg of ginger) or identical capsules of placebo (lactose). Study participants were instructed to take two capsules twice a day for three days. The first dose of study medication 

was taken 1 h before starting chemotherapy. A sample size of 35 was considered based on the method used in the similar study by Sontakke *et al*. (8). Considering 30% drop-out rate, the adjusted number of subjects to be considered for randomization was: 35 / (1 - 0.3) = 50.

The monitoring investigator dispensed either active drug or placebo according to the randomization table. After a 3-week washout period, patients were switched to the other group in a cross-over fashion. Therefore, each patient was monitored for 4 weeks. 

Study outcomes were the prevalence, severity, and duration of acute and delayed N and V. Parameters were measured using a modified standard form of Morrow Assessment of Nausea and Emesis (MANE) (11) that was given to the patients in three copies. The patients were asked to complete the forms for 24, 48 and 72 h after their chemotherapy and return them to the data collecting investigator at the hospital. 

*Data analysis*

Data were analyzed using the Statistical Package for Social Sciences (SPSS 15, SPSS Inc., Chicago, IL, USA). The McNemar and the Wilcoxon Signed Rank tests were used for the statistical analysis. The results were expressed as mean ± SD. p- values < 0.05 were regarded as significance level. 

## Results and Discussion

Fifty patients received allocated intervention. Thirteen patients excluded from the study due to non-adherence and one patient developed rash, pruritus and gastrointestinal discomfort. Thirty-six patients (10 women and 26 men) with mean ± SD age of 50.53 ± 12.21 years completed the entire 4 weeks fully blinded study protocol. The characteristics of the patients are summarized in Table 1. Data analysis revealed that the prevalence, severity, and duration of the acute and delayed nausea (Tables 2, 4 and 6) and the prevalence and severity of acute and delayed vomiting (Tables 3 and 5) were not significantly different between the ginger and placebo groups. 

**Table 1:** Patient demographics and clinical characteristics

Characteristics	Distribution
Age (years), mean ± SD	50.53 ± 12.21
Body surface area (m^2^), mean ± SD	1.79 ± 0.18
Course of chemotherapy (day), mean ± SD	4.11 ± 5.14
Dose of cisplatin (mg/m^2^), mean ± SD	103.47 ± 21.97
Sex, n (%)	
Male Female	26 (72%) 10 (28%)
Positive history of alcohol abuse, n (%)	3 (9%)
Diagnosis, n (%)	
Non-solid tumor Solid tumor Lung cancer Others	3 (8%) 33 (92%) 18 (50%) 15 (42%)
Chemotherapy regimens, n (%)	
Cisplatin + Etoposide Cisplatin + Gemcitabine Cisplatin + Docetaxel Cisplatin + Vinorelbine Cisplatin + Cyclophosphamide + Doxorubicin Cisplatin + Paclitaxel + Doxorubicin Cisplatin + 5-FU + Docetaxel Cisplatin + Pemetrexed	7 (19%) 7 (19%) 11 (31%) 4 (11%) 4 (11%) 1 (3%) 1 (3%) 1 (3%)
Susceptibility to motion sickness, n (%)	5 (15%)

**Table 2:** Prevalence of nausea in acute (day 1) and delayed phases (day 2-3).

Number of patients (%)**						Response*
Day 3		Day 2		Day 1		
Placebo	Ginger	Placebo	Ginger	Placebo	Ginger	
18 (50%)	19 (53%)	17 (47%)	20 (55.5%)	15 (42%)	19 (53%)	Control
18 (50%)	17 (47%)	19 (53%)	16 (44.5%)	21 (58%)	17 (47%)	No control

*Absence and presence of nausea was defined as control and no control. **McNemar’s test compared between ginger and placebo groups. p- value in day 1 = 0.388, in day 2 = 0.508 and in day 3 < 0.999.

**Table 3:** Prevalence of vomiting in acute (day 1) and delayed phase (day 2-3).

Number of patients (%)**						Response*
Day 3		Day 2		Day 1		
Placebo	Ginger	Placebo	Ginger	Placebo	Ginger	
8 (22%)	7 (19%)	7 (19%)	9 (25%)	9 (25%)	15 (42%)	Control
28 (78%)	29 (81%)	29 (81%)	27 (75%)	27 (75%)	21 (58%)	No control

*Absence and presence of vomiting was defined as control and no control. **McNemar’s test compared between ginger and placebo groups. p- value in day 1 = 0.070, in day 2 = 0.687 and in day 3 < 0.999

**Table 4:** Prevalence of vomiting in acute (day 1) and delayed phase (day 2-3).

p- value*	Placebo	Ginger	Response
			*Acute phase*
0.14	1.36 ± 1.91	1.75 ± 2.02	Nausea score** in day 1
			*Delayed phase*
0.31	1.5 ± 2.03	1.78 ± 1.93	Nausea score in day 2
0.73	1.47 ± 1.92	1.61 ± 1.93	Nausea score in day 3

*Absence and presence of vomiting was defined as control and no control. **McNemar’s test compared between ginger and placebo groups. p- value in day 1 = 0.070, in day 2 = 0.687 and in day 3 < 0.999

**Table 5:** Severity of vomiting in acute (day 1) and delayed phase (day 2-3).

p-value*	Placebo	Ginger	Response
			*Acute phase*
0.14	0.94 ± 1.77	1.47 ± 2.18	Vomiting score** in day 1
			*Delayed phase*
0.72	0.83 ± 1.84	1.03 ± 1.89	Vomiting score in day 2
0.78	0.92 ± 1.86	0.80 ± 1.83	Vomiting score in day 3

*p- values were calculated using Wilcoxon Signed Rank test. **Severity of vomiting reported by means of a 3-point scale: 1 = slight, 2 = moderate, 3 = severe (18).

**Table 6:** Duration of nausea in acute (day 1) and delayed phase (day 2 and 3).

p-value*	Placebo	Ginger	Response
			*Acute phase*
0.93	2.27 ± 4.88	2.18 ± 5.03	Duration** of nausea in day 1
			*Delayed phase*
0.59	5.10 ± 9.66	3.06 ± 7.61	Duration of nausea in day 2
0.82	3.80 ± 8.55	2.67 ± 6.76	Duration of nausea in day 3

*p- values were calculated using Wilcoxon Signed Rank test. **Duration ofnausea reported as total h of feeling nausea during a day (19).

The current study is one of the few randomized controlled trials (RCTs) desinged to evaluate the antiemetic effect of ginger on CINV. The results showed no significant benefical effect of ginger compared with placebo on prevalence, severity and duration of acute and delayed N and V induced by cisplatin based regimens. 

The idea that ginger may be effective for nausea and vomiting is supported by scientific evidences. Several studies have shown that ginger might be effective on N and V associated with the motion sickness (12), seasickness (13), surgury (14, 15), and pregnancy (16, 17). Animal experiments have suggested that ginger has benefical effect on N and V induced by cyclophosphamide (4) and cisplatin(5, 6).

There are limited RTCs for or against the efficacy of ginger for CINV. Pace *et al.* conducted a RCT to assess the effectiveness of ginger to prevent N and V due to the chemotherapeutic agents. They found that ginger significantly decreased a subjective nausea symptom score. The results of this study might differ from ours for several reasons including small sample size, lack of blinding, use of different outcome measures to assess the prevalence and severity of CINV, different doses and formulation of ginger, and lack of assessment of delayed CINV. Further details are unknown as only an abstract has been published concerning this study (7). Sontakke *et al.* performed a cross-over RCT and reported that ginger is as effective as metoclopramide in control of acute N and V induced by low dose of cyclophosphamide (8). Unlike our study, the 1 g dose of ginger and placebo were not co-administered with standard antiemetics, but instead ginger was compared with antiemetic medications.

The results of another cross-over RCT in gynecologic oncology patients with cisplatn based regimen have shown that 1 g of ginger has no effect in reducing acute N and V but reduces delayed N and V as effective as 40 mg metoclopramide per day (9). This study has also compared the effect of ginger with other antiemetics.

Recently, Zick *et al.* designed a well-defined RCT with broad patient sample and differenet doses of ginger. Unlike our study, this study was not designed as cross-over RCT and included the patients with different chemothepy regimens but the results were similar to ours (10). 

Our study is a cross-over RCT which offers exposure to both ginger and placebo for each patient and can omit the effect of different chemotherapy regimens. One limitation in our study was the dose of study medication. Each capsule contained 250 mg ginger, and asking patients to take more than 4 capsuls per day might result in a non-compliance. Therefore, ginger can not be administered in higher doses which may have a better impact on N and V. 

Because of the ethical issues, ginger was added to the standard antiemetics. Therefore, comparing the effect of ginger with other antiemetics was not possible in this study. 

In summary, administration of oral ginger 1g/day for 3 days did not illustrate a significant impact on CINV in patients receiving cisplatin-based regimens. Further studies with larger sample size on patients receiving moderate and low emetogenic agents, and comparing ginger with other antiemetics are needed to establish whether ginger is efficacious for CINV or not.
